# Optimization of micelle-encapsulated extremely small sized iron oxide nanoparticles as a T1 contrast imaging agent: biodistribution and safety profile

**DOI:** 10.1186/s12951-024-02699-8

**Published:** 2024-07-16

**Authors:** Minseok Suh, Ji Yong Park, Guen Bae Ko, Ji Yoon Kim, Do Won Hwang, Louis Rees, Gillian E Conway, Shareen H Doak, Hyelim Kang, Nohyun Lee, Taeghwan Hyeon, Yun-Sang Lee, Dong Soo Lee

**Affiliations:** 1 Department of Nuclear Medicine, College of Medicine, Seoul National University, Seoul, Korea; 2 Department of Nuclear Medicine, Seoul National University Hospital, Seoul, Korea; 3 Department of Molecular Medicine and Biopharmaceutical Sciences, Graduate School of Convergence Science and Technology, Seoul National University, Seoul, Korea; 4 Medical Research Center, College of Medicine, Seoul National University, Seoul, Korea; 5 Cancer Research Institute, Seoul National University, Seoul, 03080 Republic of Korea; 6 Brightonix Imaging Inc, Seoul, Korea; 7 The Interdisciplinary Program of Cancer Biology, Seoul National University, Seoul, Korea; 8 Research and Development Center, THERABEST Co., Ltd., Seoul, South Korea; 9 In Vitro Toxicology Group, Institute of Life Science, Swansea University Medical School, Swansea, Wales, UK; 10 School of Advanced Materials Engineering, Kookmin University, Seoul, Korea; 11 Center for Nanoparticle Research, Institute for Basic Science (IBS), Seoul, Korea; 12 School of Chemical and Biological Engineering, Institute of Chemical Processes, Seoul National University, Seoul, Korea; 13 Medical Science and Engineering, School of Convergence Science and Technology, Pohang University of Science and Technology (POSTECH), Pohang, Korea

**Keywords:** Iron oxide nanoparticle, ESIONs, Micelle encapsulation, MRI, T1 contrast

## Abstract

**Background:**

Iron oxide nanoparticles (IONPs) have been cleared by the Food and Drug Administration (FDA) for various clinical applications, such as tumor-targeted imaging, hyperthermia therapy, drug delivery, and live-cell tracking. However, the application of IONPs as T1 contrast agents has been restricted due to their high r2 values and r2/r1 ratios, which limit their effectiveness in T1 contrast enhancement. Notably, IONPs with diameters smaller than 5 nm, referred to as extremely small-sized IONPs (ESIONs), have demonstrated potential in overcoming these limitations. To advance the clinical application of ESIONs as T1 contrast agents, we have refined a scale-up process for micelle encapsulation aimed at improving the hydrophilization of ESIONs, and have carried out comprehensive in vivo biodistribution and preclinical toxicity assessments.

**Results:**

The optimization of the scale-up micelle-encapsulation process, specifically employing Tween60 at a concentration of 10% v/v, resulted in ESIONs that were uniformly hydrophilized, with an average size of 9.35 nm and a high purification yield. Stability tests showed that these ESIONs maintained consistent size over extended storage periods and dispersed effectively in blood and serum-mimicking environments. Relaxivity measurements indicated an r1 value of 3.43 mM^− 1^s^− 1^ and a favorable r2/r1 ratio of 5.36, suggesting their potential as T1 contrast agents. Biodistribution studies revealed that the ESIONs had extended circulation times in the bloodstream and were primarily cleared via the hepatobiliary route, with negligible renal excretion. We monitored blood clearance and organ distribution using positron emission tomography and magnetic resonance imaging (MRI). Additionally, MRI signal variations in a dose-dependent manner highlighted different behaviors at varying ESIONs concentrations, implying that optimal dosages might be specific to the intended imaging application. Preclinical safety evaluations indicated that ESIONs were tolerable in rats at doses up to 25 mg/kg.

**Conclusions:**

This study effectively optimized a scale-up process for the micelle encapsulation of ESIONs, leading to the production of hydrophilic ESIONs at gram-scale levels. These optimized ESIONs showcased properties conducive to T1 contrast imaging, such as elevated r1 relaxivity and a reduced r2/r1 ratio. Biodistribution study underscored their prolonged bloodstream presence and efficient clearance through the liver and bile, without significant renal involvement. The preclinical toxicity tests affirmed the safety of the ESIONs, supporting their potential use as T1 contrast agent with versatile clinical application.

**Supplementary Information:**

The online version contains supplementary material available at 10.1186/s12951-024-02699-8.

## Background

Iron oxide nanoparticles (IONPs) are one of the few nanomaterials that have received FDA clearance for clinical applications. These applications span a wide range, including tumor-targeted imaging [1], magnetic hyperthermia therapy [2, 3], drug delivery systems [4, 5], and live-cell tracking through cell labeling [6, 7]. Traditionally, IONPs have been utilized in the clinical field as magnetic resonance imaging (MRI) T2 contrast agents due to their superparamagnetic properties [8–10]. The first generation IONPs, such as ferumoxide and ferucarbotran, were primarily used as liver imaging agents [11–13]. However, their relatively large size (dH ∼ 50-200nm) led to rapid clearance from the bloodstream and prolonged retention in the liver, limiting their clinical utility. Consequently, ultrasmall superparamagnetic IONPs (USPIO) with a hydrodynamic size of 20–50 nm were introduced as a next-generation imaging agent, designed to evade reticuloendothelial system (RES) uptake and ensure longer blood circulation [10]. This advancement allowed for their application in a variety of clinical contexts, including lymph node [14–17], plaque [18], and central nervous system macrophage imaging [19]. Despite these developments, T2 contrast agents are not without their limitations, such as the ‘blooming effect’ [20, 21], leading to the discontinuation of several iron oxide imaging agents in clinical settings across the United States and most of Europe [22].

Emerging from the lineage of IONPs, extremely small-sized IONPs (ESIONs) represent a pivotal shift towards enhancing the versatility and applicability of IONPs in clinical settings. Distinct from their larger counterparts, ESIONs have a hydrodynamic size smaller than 5 nm, which significantly reduces uptake by the reticuloendothelial system, thereby extending their circulation time within the bloodstream [20]. Furthermore, this size reduction has transitioned their utility toward serving as T1 contrast agents in MRI, marked by a high longitudinal relaxivity (r1) and a low relaxivity ratio (r2/r1), desirable traits for effective T1 contrast enhancement [20, 23–26]. This capability could enhance clinical diagnostics, providing more precise, clearer imaging for a range of conditions including cancer, cardiovascular diseases, and neurological disorders, thereby facilitating early diagnosis and treatment.

In light of these developments, our research focuses on the micelle encapsulation method for hydrophilizing nanomaterials, a technique our group has recently refined to facilitate the rapid and straightforward production of multifunctional nanoparticles under mild conditions [27]. This study aims to further refine and scale-up the process of micelle encapsulation for ESIONs, enhancing their compatibility and effectiveness as T1 contrast agents. Additionally, our in vivo biodistribution and toxicity studies in rats seek to validate the safety and efficacy of these nanoparticles, paving the way for their clinical translation.

## Methods

### Optimization for scale-up process of micelle encapsulated ESIONs

**Fig. 1:**
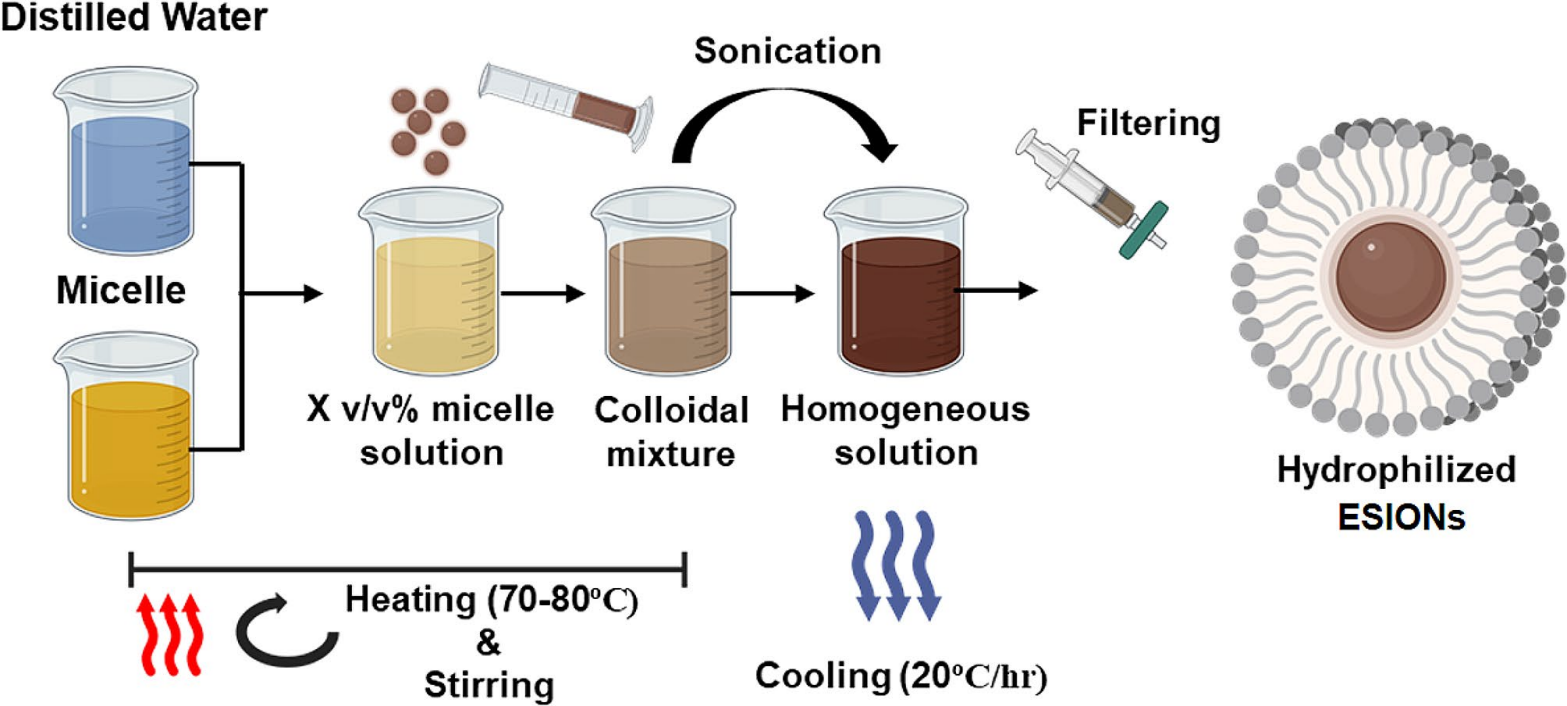
Schematic procedure of hydrophilic conversion using micelle encapsulation method

The schematic procedure of micelle encapsulated ESIONs synthesis is summarized in the Fig. 1. In this study, we used 3 nm core ESIONs synthesized via the thermal decomposition method, described in the previous report [28]. These nanoparticles were initially dispersed in chloroform, setting the stage with an initial iron (Fe) concentration of 20 mg/ml.

To determine the optimal hydrophilic concentration for dispersing the ESIONs in an aqueous solution, the hydrophilic conversion process was performed using different concentrations of Tween60 and Tween80. Initially, solutions of Tween60 and Tween80 at 2.5 v/v%, 5 v/v%, 10 v/v% and 20 v/v% were prepared using 1.5 mL of iron oxide (20 mg/mL) as a standard. The hydrophilic conversion process was systematically undertaken by heating distilled water mixed with Tween60 and 80 on a hot plate set between 70 and 80 °C. Maintaining this temperature, different concentrations of Tween60 and 80 were gradually introduced into 12 mL of distilled water with constant stirring, followed by the addition of the iron oxide solution. This mixture was then sonicated until a cloudy solution formed, thereafter continuing with stirring on the hot plate to facilitate solvent removal.

Subsequent to this preparation, the upper layer of each sample, stored under cold conditions, was collected and subjected to filtration using a syringe filter (0.22 μm), ensuring purity and uniformity. The samples were then stored in a cool environment. The practicality of this purification method was further corroborated using Opti-Prep density gradient separation as outlined in Supplementary Fig. 1a, offering a straightforward approach to segregate non-hydrophilic nanoparticles from their hydrophilic counterparts and the micelles [29]. The yield and hydrodynamic size of micelle-encapsulated ESIONs were compared based on different surfactant concentrations.

Moving forward to the scale-up experimentation, we adhered to the original reaction blueprint while expanding the reaction volume to enhance the yield of ESIONs proportionally. Table 1 intricately presents the composition for a 40 mL micelle solution used in encapsulating 3 nm ESIONs. In this formulation, the solution contains 57 mg of Fe_2_O_3_ out of a total dry weight of 100 mg, adhering to the core mass fraction (f = M_core/M_total) of 0.57, as established from previous research [28]. The remaining 43 mg is composed of pure oleic acid, corresponding to 0.16 mmole. The reaction mixture of 40 mL micelle solution was formulated by combining 4 mL of Tween60 with 36 mL of solvent (10% v/v concentration), which equates to 3.18 mmole of Tween60 within the solution. This setup preserves a constant molar ratio of oleic acid to Tween60 ratio at 20. In the scale-up from 40 mL to 400 mL, we maintained the oleic acid to Tween60 ratio across both scales. The scale-up to 400 ml was strategically planned to ascertain a scalable process capable of producing up to 800 mg of nanoparticles per day by a single individual.

**Table 1:** Chemical profile of micelle encapsulation method for 3 nm ESIONs

	Mw	Density	Concentration	mle scale
Tween60 micelle	1311.7 g/mol	1.044 g/mL	4 mL/40 mL	3.18 mmole/40 mL
Oleic acid	282.46 g/mol	ESIONs 20 mg/mL x 5 mL = 100 mg of ESIONs	57 mg ESIONs + 43 mg Oleic acid	0.16 mmole/43 mg Oleic acid
ESIONs core	95 kDa			0.000589 mmole/57 mg ESIONs core

ESIONs, extremely small-sized iron oxide nanoparticles

Throughout this phase, we undertook a total of five distinct experiments at the 40 mL scale to ensure repeatability and reliability. Each of these trials allowed us to precisely calculate the theoretical weights of the nanoparticle cores and the sole iron content at each stage. Subsequently, the yields were evaluated through the quantification of the iron content using a colorimetric reagent, underlining the robustness and efficiency of our optimized scale-up process.

### Long term stability and agglomeration state of micelle encapsulated ESIONs

To assess the practical application of our synthesized micelle-encapsulated ESIONs, two experiments were conducted to evaluate their long-term stability and agglomeration potential in liquid state.

The first experiment focused on monitoring the ESIONs suspension at room temperature to detect any potential aggregation. This assessment was conducted over a period of 30 days, with daily measurements to track any changes in the size of the nanoparticles. Additionally, we examined the response of the ESIONs to different pH levels to understand their behavior under various physiological conditions. The hydrodynamic diameter and size distribution were measured using a Zetasizer Nano ZS90 DLS system (Malvern Instruments Ltd, Worcestershire, UK).

The second experiment was designed to simulate the nanoparticles’ exposure to an in-vivo-like environment, particularly focusing on the impact of the protein corona, which could induce aggregation. In this scenario, we used human serum for the experimental group and phosphate buffered saline (PBS) for the control group, to reflect the basic physiological conditions the ESIONs might encounter. For this, 0.1 mL of the ESIONs solution was mixed with 0.9 mL of either human serum or PBS. This mixture was then kept at body temperature (36.5 °C) and agitated continuously for 24 h. Following this period, we collected samples from the mixtures for analysis. The morphology of the nanoparticles after this incubation was analyzed using transmission electron microscopy (TEM).

### R1 and R2 measurements for checking the relaxivity of ESIONs

The T1 and T2 relaxation times of micelle encapsulated ESIONs and radiolabeled ESIONs were measured using a 1.41 T minispec mq 60 NMR Analyzer (Bruker, Germany) at 37 °C. Relaxivity values were calculated via linear least-squares fitting of 1/relaxation time (s^− 1^) vs. the iron concentration (mM).

### Radiolabeling of micelle encapsulated ESIONs

For radio-isotopic labeling, we used the method from previous studies employing ligands with a long alkyl chain. Specifically, 2-(p-isothiocyanatobenzyl)-1,4,7-triazacyclononane-1,4,7-triacetic acid [NOTA]-C18 was used for incorporation into micelles (Supplementary Fig. 1b). We prepared NOTA-micelle by mixing 2 mol% of NOTA-C18 with 10 v/v% Tween60 micelles during the micelle preparation stage, based on the optimized micelle encapsulated ESIONs. Subsequently, micelle encapsulated ESIONs were formed using the same process.

For the radiolabeling process, a vial containing the radioisotope ^64^Cu was dried using dry N_2_ gas. Once fully dried, 500 µL of 1 M sodium acetate buffer (pH 5.3) was added to adjust the pH to 5.0. Then, 1 mL of the encapsulated ESIONs was introduced into the vial and allowed to incubate at room temperature for 30 min to complete the radiolabeling.

Following this step, the mixture underwent a purification and neutralization process where the buffer and any unlabeled radioisotopes were separated using an Amicon filter (Amicon Ultra-0.5, 100 kDa, Merck Millipore) set at 10,000 rpm and 25 °C for 2 min.

The efficiency and stability of the radiolabeling were assessed using radio-instant thin layer chromatography-silica gel (radio-ITLC-SG), with citric acid serving as the mobile phase (retardation factor (Rf) of free radioisotope = 0.9-1.0; Rf of radiolabeled ESIONs = 0.0-0.1). The results from the ITLC-SG were documented using a TLC scanner (AR-2000, Bioscan, U.S.A.).

To evaluate the storage stability of the radiolabeled ESIONs, samples were stored in phosphate-buffered saline (PBS) at room temperature and checked at baseline, 1, 4, and 24 h. We also examined the stability of these ESIONs in human serum. For this, 0.1 mL of radiolabeled ESIONs were combined with 1 mL of filtered human serum and incubated at 37 °C. The radiochemical purity of these mixtures was then assessed at the same time intervals.

Furthermore, we compared the hydrodynamic diameter and relaxivity between the micelle encapsulated ESIONs and the radiolabeled versions to determine any significant changes post-labeling.

### Biodistribution study

The animal experiments were approved by the Institutional Animal Care and Use Committee at Seoul National University. Specific pathogen-free, 6-week-old male BALB/c mice were used, obtained from Orient Bio (Seongnam, Korea).

For the image-based biodistribution study, we utilized the SimPET system from Brightonix Imaging (Seoul, South Korea), which combines a 1-T permanent magnet-based MRI (M7; Aspect Imaging, Shoham, Israel), characterized by a peak sensitivity of 4.2% and a center volumetric resolution of 0.63 mm³ [30, 31]. The mice received an injection through the tail vein of ^64^Cu-ESIONs with an iron concentration of 5 mgFe/kg (11.25 ± 0.7 Mbq/200 µl). Pre-injection, a 3-dimensional (3D) T1-weighted gradient echo (GRE) MRI scan was performed (TR/TE, 9/2.8 ms; Flip angle, 45 degrees).

Dynamic PET imaging was acquired for 56 min right after the injection of radiolabeled ESIONs and later reconstructed to 8 sequential images with 7 min interval. Simultaneously, 3D T1-weighted GRE MRI images were acquired with the same 7 min interval. PET/MRI images of serial time points were further acquired at 2, 3, 4, 24, 48, and 72 h after injection for 7 min. Acquired images were reconstructed using a 2D ordered-subsets expectation maximization algorithm with scatter and decay correction. The volume of interest (VOI) was manually drawn over the heart, reflecting the blood pool activity, on MRI images of mice acquired at different time points. The activity (Bq) measured in the heart was normalized to the total injected dose of each radiotracer and divided by the volume to obtain percentage injected dose per volume (%ID/ml). The PET image-based blood pool biodistribution was plotted as a function of time to generate time-activity curves (TACs).

For the ex vivo biodistribution study, mice were sacrificed at serial time points of 5 min, 1, 4, and 24 h (*n* = 3, respectively, total = 12), after injection of ^64^Cu-ESIONs (dose = 1 µCi/100 µL). Urine and feces were collected from 0 to 4 h and from 4 to 24 h. Radioactivity of blood, major organs (heart, lung, liver, spleen, stomach, intestine, and kidney) and excreta (urine and feces) was counted using a gamma scintillation counter (DREAM r-10, Shinjin Medics Inc., South Korea).

To assess the in-vivo stability, we tested the radiochemical purity of radiolabeled ESIONs in blood and feces samples at various times post-injection, using ITLG-SG.

### Dose-dependent MRI signal change

For the quantitative evaluation of signal enhancement in the MRI, signal intensity ratio (SIR) was calculated as following.

$$\:SIR=\frac{\text{p}\text{o}\text{s}\text{t}-\text{c}\text{o}\text{n}\text{t}\text{r}\text{a}\text{s}\text{t}\:\text{s}\text{i}\text{g}\text{n}\text{a}\text{l}\:\text{i}\text{n}\text{t}\text{e}\text{n}\text{s}\text{i}\text{t}\text{y}}{\text{p}\text{r}\text{e}-\text{c}\text{o}\text{n}\text{t}\text{r}\text{a}\text{s}\text{t}\:\text{s}\text{i}\text{g}\text{n}\text{a}\text{l}\:\text{i}\text{n}\text{t}\text{e}\text{n}\text{s}\text{i}\text{t}\text{y}}$$

In the phantom study, ESIONs were diluted into 5 different concentrations. At each step, ESIONs were diluted to half of the previous concentration starting from 1 mM. The diluted ESIONs and normal saline, as a control, were filled into plastic vials and embedded into a plastic container that has multiple crafted holes for vial insertion. The container was placed in a perpendicular direction to the main magnetic field and the same PET/MRI protocol, as done in the biodistribution study, was applied. Circular VOI was drawn over each vial inserted holes for the quantification.

Time-dependent change of blood pool MRI signal was evaluated in vivo using ESIONs with 3 different concentrations (2.5 mgFe/kg, 5 mgFe/kg and 10 mgFe/kg). Ten sequential images of 3D T1-weighted GRE with 3.5 min interval were acquired right after the injection of ESIONs with different concentration. An additional 1.5-hour MRI image was acquired. For the quantitative evaluation of vascular contrast enhancement, SIR was quantified using the manually drawn VOI of the aorta.

### Toxicity evaluation of micelle encapsulated ESIONs

For the purposes of in vitro toxicological studies, the human hepatocellular carcinoma cell line, HepG2, was selected due to its detection sensitivity towards hepatotoxic drugs. HepG2 cells (ATCC HB-8065) were cultured as a monolayer in Dulbecco’s Modified Eagle Medium (DMEM) supplemented with 4.5 g/L d-glucose and l-glutamine (GIBCO, Paisley, UK), 10% fetal bovine serum (FBS), and 1% penicillin/streptomycin antibiotic (GIBCO, Paisley, UK). The ESIONs stock was then diluted using DMEM to the required working concentrations for a 24-hour exposure. The cell suspension from the day of seeding was replaced with 3 mL of the desired working concentration of ESIONs (0–960 µg/mL) and incubated for 24 h at 37 °C/5% CO2. Cytotoxicity was assessed using the Trypan Blue exclusion assay, where cells were stained with Trypan Blue dye and counted using a hemocytometer. Viable cells exclude the dye and remain unstained, while non-viable cells take up the dye, allowing for the determination of cell viability.

Preclinical toxicity evaluations were conducted at Biotoxtech Co., Ltd. (Cheongwon, Korea), adhering to the Good Laboratory Practice Regulations set by the Korean Ministry of Food and Drug Safety on 21-Nov-2018. The study was organized into four groups: three dosage levels at 2.5, 5, and 25 mg/kg, and one control group receiving normal saline. Each group included five male and five female animals and was divided into an interim kill group (necropsy at 2 days post-administration) and a main test group (necropsy at 14 days post-administration). We assessed general health, behavior, appearance, body weight, food consumption, ophthalmologic health, and urine output.

Before necropsy, all animals were fasted for at least 18 h. They were then anesthetized using isoflurane, and blood samples were collected from the abdominal aorta. For hematological analysis, approximately 1 mL of blood was placed into EDTA tubes, and a complete blood count was conducted using a hemocytometer (XN-V, SYSMEX, Japan). Coagulation tests were performed using around 2 mL of blood, mixed with 3.2% sodium citrate, centrifuged to obtain plasma, and then analyzed for prothrombin time and activated partial thromboplastin time using a coagulation time analyzer (Coapresta 2000, SEKISUI, Japan). Clinical biochemical parameters were measured using the remaining blood, after centrifugation, with assessments conducted by a biochemistry analyzer (7180, HITACHI, Japan) and an electrolyte analyzer (EasyLyte, MEDICA, U.S.A.).

Following blood collection, mice were euthanized on days 2 and 14 post-administration, and a detailed dissection was performed for macroscopic examination. The collected organs and tissues were then preserved in a 10% neutral buffered formalin solution. After fixation, samples were processed into paraffin sections for subsequent histological examination.

## Results and discussions

### Optimization of encapsulation-based scale-up processes

**Fig. 2:**
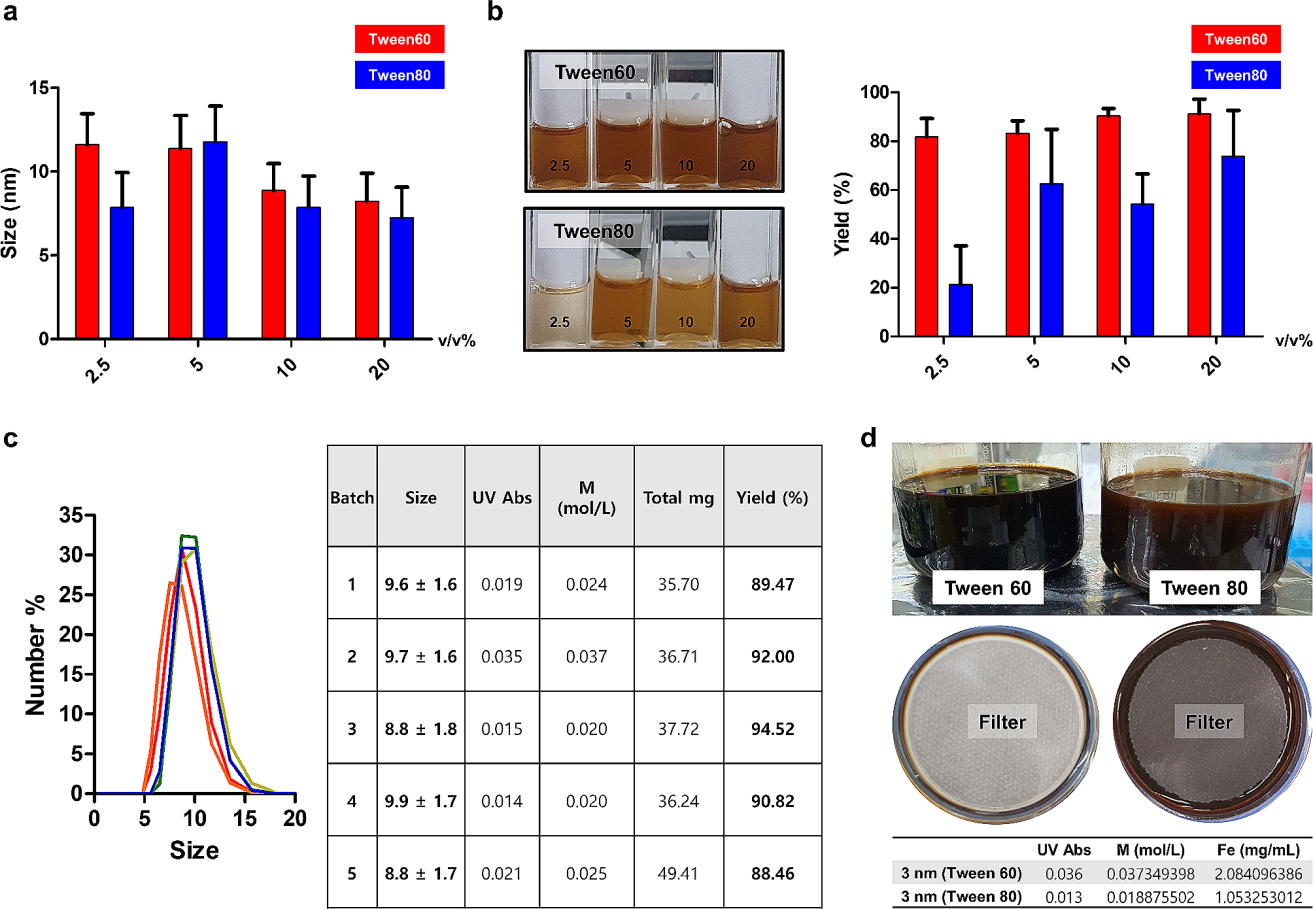
**a,** DLS size of each v/v % of micelle concentration using Tween 60 or Tween 80 after encapsulation of 3 nm extremely small sized iron oxide nanoparticles (ESIONs). **b,** The apparent color of the ESIONs aqueous solutions after purification and the corresponding yield graphs at each v/v% concentration were examined. **c,** Verification of reproducibility for 5 batch repeat experiments using 100 mg of iron oxide and a 40 mL micelle solution under established conditions. **d,** High-capacity production of hydrophilic nanoparticles obtained through a 400 mL scale reaction and the yield differences observed during large-scale filtration. Tween 60 exhibited twice the yield compared to Tween 80, consistent with the trend observed in Fig. 2b at the 40 mL scale

Our study identified optimal conditions for large-scale production and dispersion of 3 nm ESIONs in an aqueous solution, finding that using Tween60 at a 10% v/v concentration was the most effective approach. It was noted that increasing the concentration of the hydrophilic agents from 2.5% v/v to 10% v/v led to a decrease in particle size, suggesting insufficient hydrophilic material at lower concentrations (Fig. 2a). However, size changes were not significant between 10% v/v and 20% v/v, indicating that 10% v/v concentration is sufficient for micelle-mediated hydrophilization. Furthermore, Tween60 showed consistently higher yield efficiency compared with Tween80 (Fig. 2b**).**

The scaling process was effectively optimized, achieving a consistent yield over 88% across batches with hydrophilic conversion, as shown by the DLS sizes ranging from 8.835 to 9.605 nm with a uniform standard deviation (Fig. 2c). The scale-up to 400 mL, a ten-fold increase from the original 40 mL scale, demonstrated Tween60’s superior hydrophilic conversion efficiency compared to Tween80 (Fig. 2d), achieving approximately 500 mg of water-soluble nanoparticles from a theoretical 570 mg of ESIONs. Given that the entire process takes less than half a day and yields an average of 90%, this efficiency enables researchers to achieve gram-scale hydrophilic conversion within a day.

This contrasts with traditional hydrophilicity enhancement methods, such as ligand substitution or surface coating [32, 33]. Substitution involves exchanging the hydrophobic ligands on the surface of nanoparticles with hydrophilic substances, whereas coating entails enveloping the nanoparticle surface with hydrophilic materials such as polymers or silica. The commonly employed substitution method involves utilizing substances like PO-PEG to perform ligand substitution in organic solvents, followed by re-dispersion in water to change the solvent phase to a dispersing medium [34]. This method requires a substantial amount of PO-PEG for hydrophilization and entails additional purification steps during functionalization, posing challenges to yield and process stability.

Moreover, the inherent complexity of these multi-step reactions often leads to issues such as nanoparticle aggregation due to the polarity changes in terminal functional groups, leading to batch inconsistency. This typically results in a significant hydrodynamic radius increase post-hydrophilization. Additionally, using non-FDA-cleared agents for hydrophilization raises biocompatibility and safety concerns for the resulting nanoparticles [35].

In contrast, our method enables simultaneous surface modification and hydrophilization, facilitating the straightforward addition of disease-targeting molecules, chelating agents, and fluorescent probes [16, 27, 36]. This approach allows for immediate adjustments if particle aggregation occurs, with surface modifications completed in a single-step reaction to ensure high yields of the final nanoparticles. Additionally, by applying combinatorial chemistry principles, we can introduce a variety of functional groups either singly or in combination, improving the nanoparticles’ disease-targeting and labeling efficiency. Notably, this process utilizes Tween60, an FDA-cleared and biocompatible hydrophilic agent, ensuring stability and precision in encapsulating functional groups within micellar forms. By harnessing specific molecules for disease targeting, such as for cancer or cardiovascular conditions, and incorporating diagnostic fluorescent probes, our method can enhance the precision and efficiency of treatments with reduced side effects.

### Long term stability and agglomeration state of micelle encapsulated ESIONs

**Fig. 3:**
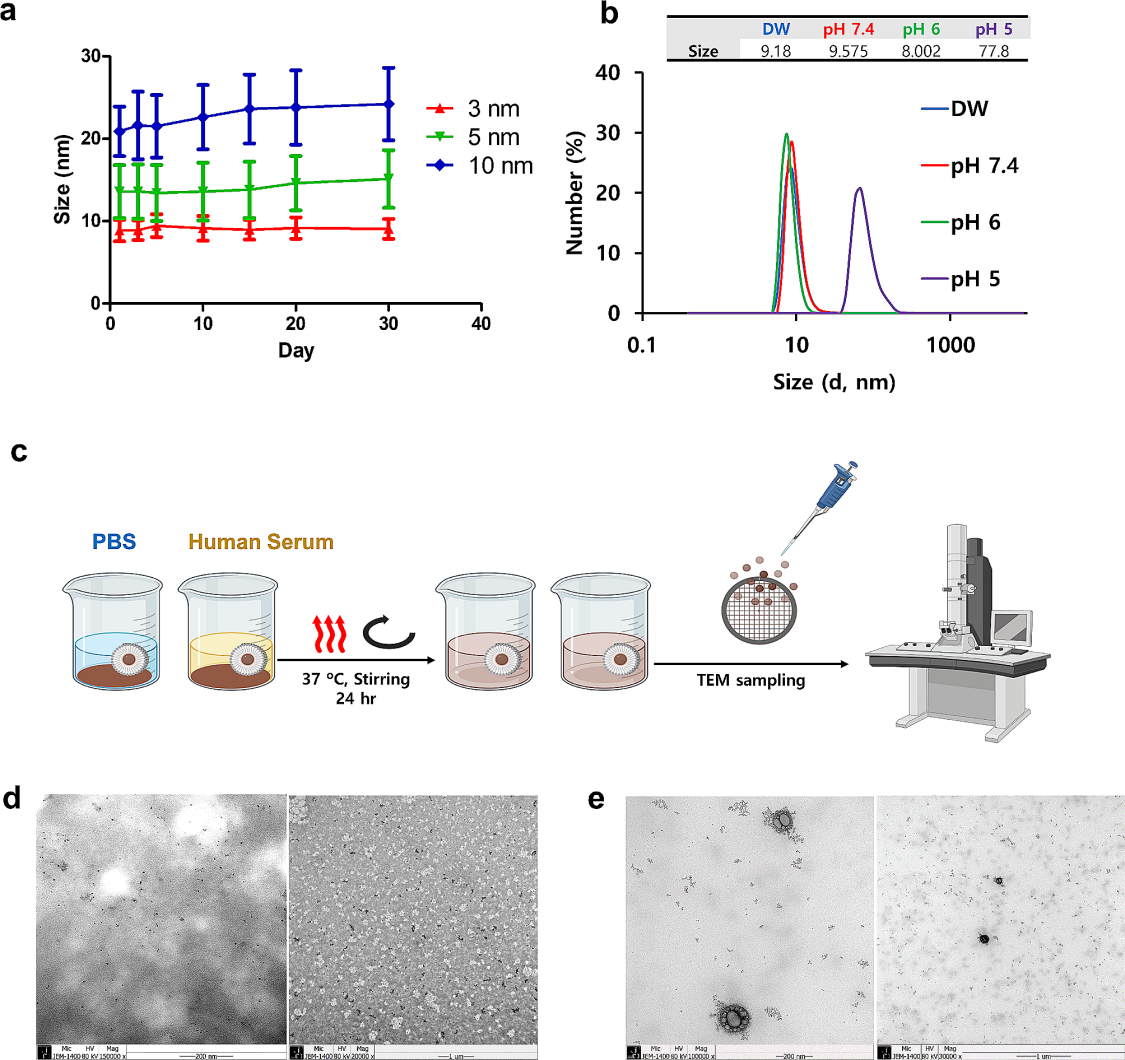
**a,** Measurement of sizes through Dynamic Light Scattering (DLS) for hydrophilic 3, 5, and 10 nm nanoparticles during 30 days of room temperature storage. **b,** Evaluation of aggregation behavior of 3 nm hydrophilic iron oxide nanoparticles as a function of pH, monitored through changes in DLS size measurements. **c,** A schematic representation of the evaluation method for nanoparticle aggregation under in vivo temperature and serum conditions, incorporating hydrophilic nanoparticles. **d,** TEM image of 3 nm nanoparticles under PBS conditions. **e,** TEM image of 3 nm nanoparticles under human serum conditions

The long-term stability and aggregation state of micelle-encapsulated ESIONs for core sizes of 3 nm, 5 nm, and 10 nm were assessed. All sizes were synthesized using the same method as that developed for the 3 nm particles. The hydrophilic ESIONs with a size of 3 nm showed minimal size deviation during storage **(**Fig. 3a**).** In contrast, larger nanoparticles, those with sizes of 5 nm and above, displayed more significant size changes, likely due to sedimentation or increase in size affecting the hydrophilic agent’s coating efficiency during storage. This variation suggests a diminished effectiveness of the hydrophilic coating with increasing particle size, emphasizing the necessity to optimize particle size and coating parameters, especially for particles over 5 nm, to ensure their stability for prolonged periods [16, 36].

The stability of these nanoparticles under various pH conditions was also investigated, creating environments with pH levels of 7.4 (neutral), 6, and 5. The size stability of nanoparticles in these solutions confirmed that they remained more stable and less prone to agglomeration in neutral conditions compared to acidic environments, as evidenced by substantial agglomeration in the acidic pH 5 setting (Fig. 3b). This underscores the critical influence of pH on nanoparticle stability and dispersion.

Furthermore, to simulate real blood conditions, the nanoparticles’ response to albumin was tested. TEM images after exposure to PBS and HSA show that while there was slight aggregation, the nanoparticles generally stayed well-dispersed (Fig. 3c-e). This behavior, particularly following hydrophilic conversion with high-capacity PEG, suggests enhanced stability against agglomeration in conditions that mimic the bloodstream, affirming their potential use in clinical settings.

### Relaxivity of micelle encapsulated ESIONs

The relaxivity characteristics of micelle-encapsulated ESIONs, particularly those with a 3 nm core size, were thoroughly evaluated. These nanoparticles demonstrated an r1 relaxivity of 3.43 mM^− 1^s^− 1^ and an r2/r1 ratio of 5.36 (Supplementary Fig. 2c). The effectiveness of T1 contrast agents is typically gauged by their r1 relaxivity values; the higher the r1, the better the agent at enhancing T1 contrast. Most clinically utilized contrast agents fall within the relaxivity range of 3.7 to 5.9 mM^− 1^s^− 1^ at 1.5T [37]. The r1 relaxivity of 3.43 mM-1s-1 measured at 1.41T is modestly lower compared to typical gadolinium-based agents. Additionally, relaxivity tends to decrease with increasing magnetic field strength. Studies indicate that the change in r1 relaxivity of ESIONs from 1.5T to 3T is approximately a 10–30% decrease [38]. Therefore, it is anticipated that the r1 relaxivity of our encapsulated ESIONs might be around 2 to 3 mM − 1s − 1 at 3T. While this is lower than traditional agents, it remains within a clinically relevant range. Additionally, the r2/r1 ratio of 5.36 for ESIONs, while higher than the typical 1 to 2 range for Gadolinium-Based Contrast Agents (GBCAs), still underscores the suitability of our materials more as T1 agents than T2. This is especially notable as IONPs, typically employed as T2 agents, have significantly higher r2/r1 ratios. Thus, the relaxivity profile emphasizes the potential of micelle-encapsulated ESIONs as effective T1 contrast agents.

### In vivo biodistribution of micelle encapsulated ESIONs

There was no significant change in size and relaxivity after the radiolabeling procedure (Supplementary Fig. 2). Labeling efficiency of ^64^Cu-ESIONs was over 95% and remained stable until 24 h in PBS solution at room temperature (92.4%) and in human serum at 37 °C (77.1%) (Supplementary Fig. 3).

**Fig. 4:**
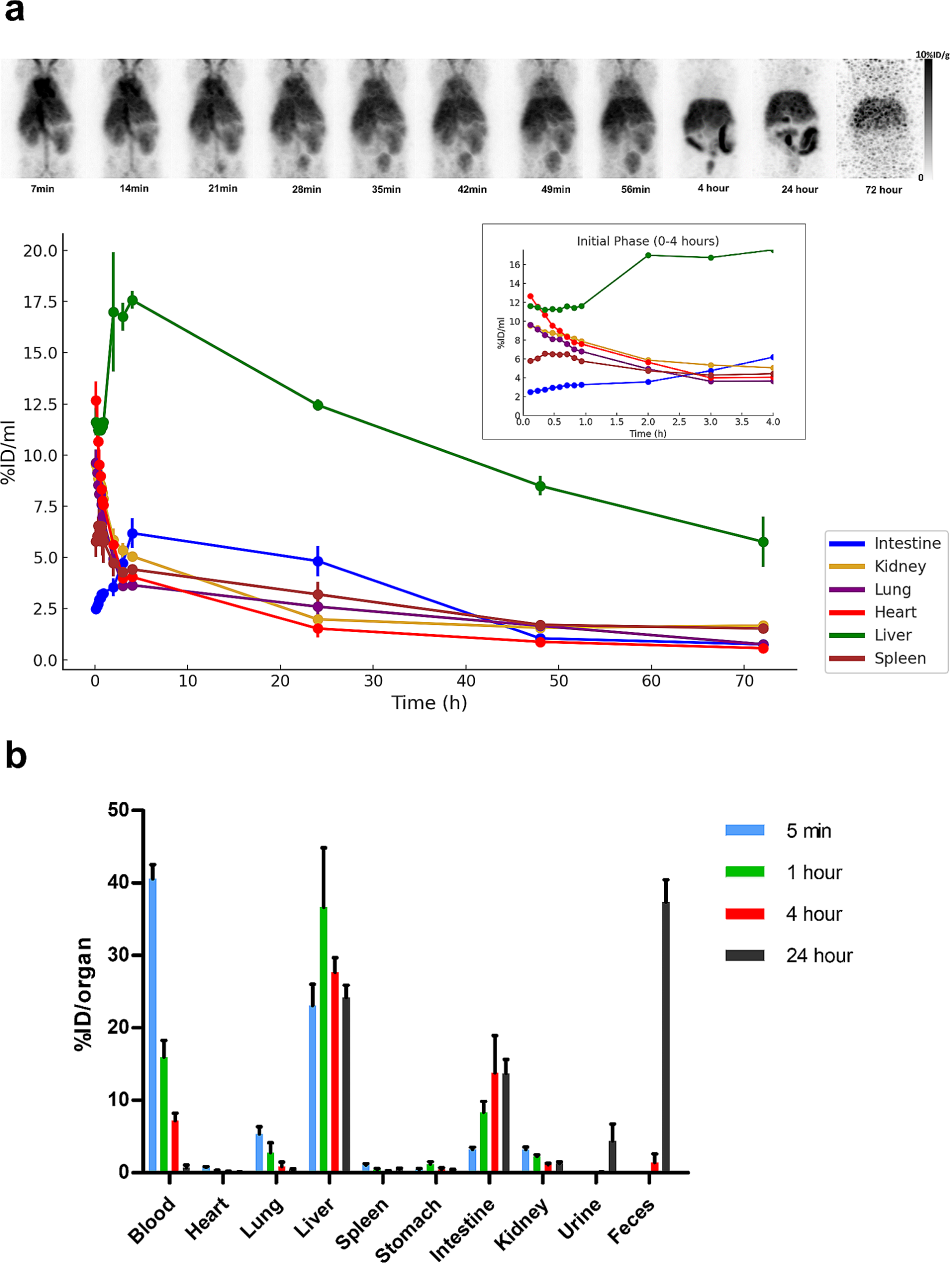
**a,** Decay-corrected MIP (Maximum Intensity Projection) images in BALB/c mice at different time points after intravenous injection of ^64^Cu-ESIONs were shown and quantitatively analyzed. Major organ uptake at serial time points was obtained and was expressed as mean ± SD (%ID/ml). **b,** Ex vivo biodistribution of BALB/c mice after intravenous injection ^64^Cu-ESIONs was quantitatively analyzed. Major organ uptake at serial time points was obtained and was expressed in units of %ID/organ

Early imaging showed the ^64^Cu-ESIONs predominantly within blood pool organs such as the heart, lungs, and kidneys, as illustrated in Fig. 4a. The time-activity curve of the heart indicated a biphasic decrease in radioactivity, with an initial half-life of about 62 min, shifting to a slower decline phase with a half-life of approximately 12.8 h. As time progressed, the activity within the blood pool diminished, whereas liver and intestinal uptakes peaked within the first four hours before gradually decreasing over 72 h. The ex vivo data, reported as percentage injected dose per organ (%ID/organ), show rapid clearance from blood, lung, and kidney, with significant radioactivity still present in the blood at one and four hours post-injection, detailed in Supplementary Tables 1 and Fig. 4b. Notably, fecal accumulation accounted for 38.77 ± 3.11% of the total injected dose over 24 h.

Compared to previous studies on IONPs, our micelle-encapsulated ESIONs demonstrated extended circulation time, a notable improvement over the rapid clearance rates observed in smaller [26] or larger [39–42] IONPs. Besides the short blood circulation time, the degradation and excretion process in the RES is significantly slow for larger IONPs, which can take from months to years [43, 44]. The size of micelle encapsulated ESIONs in this study (9.35 nm) seems to be optimal as an effective blood pool imaging agent by minimizing uptake in RES such as Kupffer cells and avoiding renal clearance so that it can remain in the blood pool for a long time. Extended vascular retention mirrors the benefits of gadolinium-based blood pool contrast agents, allowing for high-resolution, time-resolved imaging crucial for diagnosing vascular abnormalities, evaluating cardiac function, and identifying tumor angiogenesis [45, 46]. Additionally, extended circulation time may facilitate, enhanced permeability and retention (EPR) effect, selective accumulation in regions with compromised endothelial integrity, leading to superior differentiation between diseased and normal areas [47, 48]. Furthermore, the surface modification and hydrophilization characteristics of ESIONs facilitate easy integration of disease-specific targeting molecules, chelating agents, and fluorescent markers. This flexibility permits the broader application of ESIONs across different medical fields, augmenting their utility as multifunctional agents in targeted treatments and diagnostic processes.

To ascertain the composition and in vivo stability of excreta, we estimated the radiochemical purity of blood and feces. Blood samples, collected at various times (5 min, 1 h, and 4 h), and showed identical peaks to the baseline ^64^Cu-ESIONs, as depicted in Supplementary Fig. 4a. Additionally, stool content from the large bowel, when mixed with normal saline, exhibited a high radiochemical purity of 81% for ^64^Cu-ESIONs, as illustrated in Supplementary Fig. 4b. Urine collected one hour after an injection of 64Cu-ESIONs revealed that most of the radioactivity was detected as a disintegrated form (Supplementary Fig. 4c). Given the high radiochemical purity in the blood and the presence of mostly disintegrated form in urine, it is speculated that radiolabeled ESIONs will be released through urine as soon as it is disassociated. Since, the urinary excretion was minimal (%ID: 4.45%, at 24 h), the effect of disintegration in our study might be negligible.

In the study rapid hepatobiliary excretion of micelle-encapsulated ESIONs was noted. Seo et al. have shown that similar micelle-encapsulated upconverting nanoparticles (UCNPs) are also eliminated via hepatobiliary routes while maintaining their intact form, as confirmed using PET and TEM imaging [49]. This suggests that micelle encapsulation might enhance the hepatobiliary clearance of nanoparticles, although the precise biological mechanisms remain unclear. It is hypothesized that the encapsulation reduces recognition by the reticuloendothelial system (RES), leading to the efficient uptake of micelle-encapsulated ESIONs by hepatocytes. Heine et al. have demonstrated that, unlike polymer-coated nanoparticles that primarily accumulate in liver sinusoidal endothelial cells, micelle-encapsulated nanoparticles are absorbed by both hepatocytes and Kupffer cells [50]. This distribution varies between normal and LDL receptor knock-out transgenic mice, indicating that LDL receptors and apolipoprotein E (ApoE) significantly influence the hepatic uptake of these nanoparticles.

### Dose-dependent MRI signal change

**Fig. 5:**
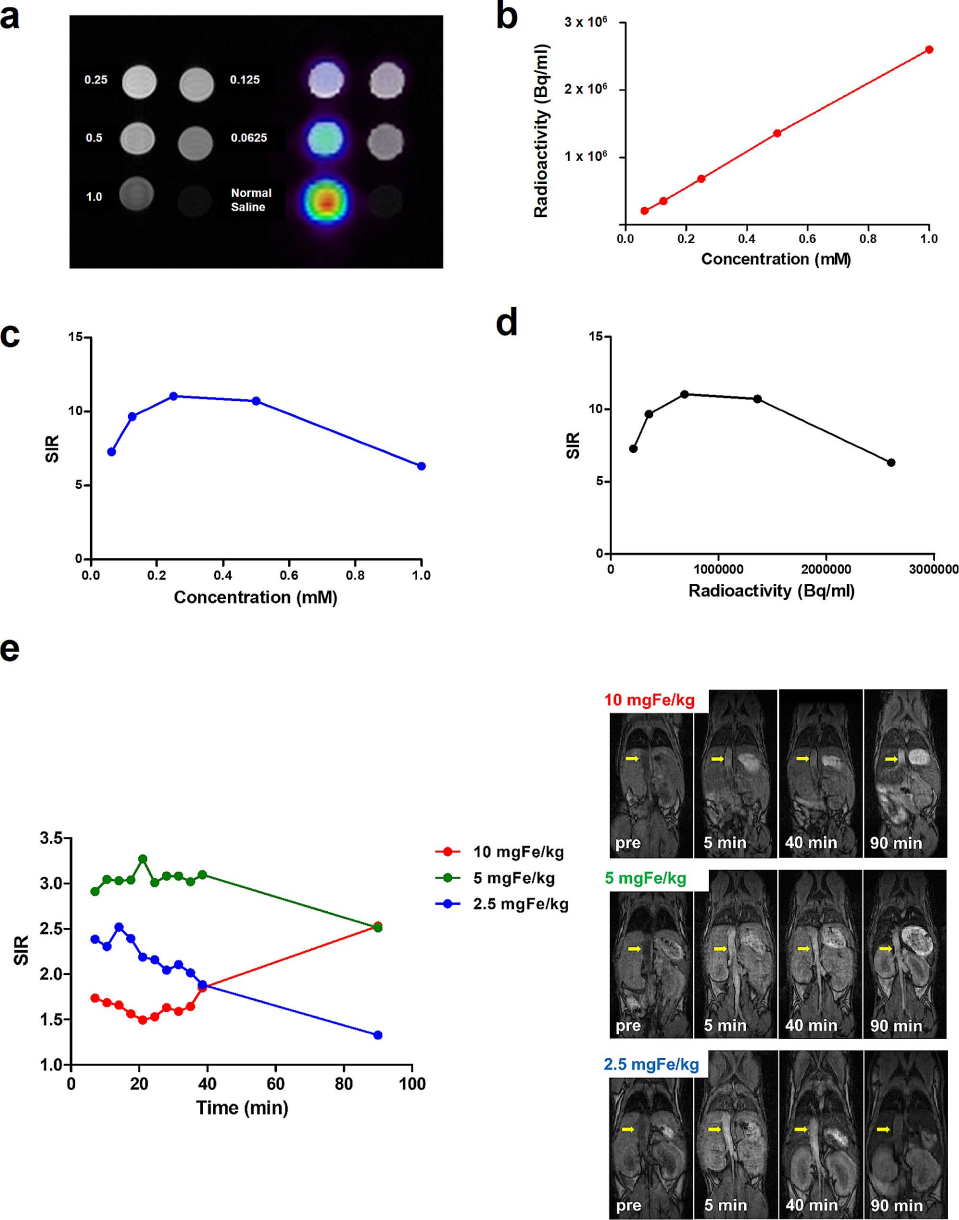
**a,** Concentration-dependent change of PET and MRI signal was compared using phantom of sequentially diluted ^64^Cu-ESIONs. **b,** Relationship between ESIONs concentration and PET signal, **c,** concentration and MRI signal, **d,** PER and MRI signal is plotted. MRI signal intensity was expressed as signal intensity ratio (SIR) and PET as Bq/ml. **e,** Time activity curve of SIR was compared between 3 different concentration (10 mgFe/kg, 5 mgFe/kg and 2.5mgFe/kg) of ESIONs

Phantom study showed that dose-dependent change of SIR was non-linear (Fig. 5a). Specifically, SIR increased with lower concentrations, but decreased as the concentration rose. In vivo assessments using three different ESIONs concentrations showed varied time-dependent changes in blood pool MRI signal (Fig. 5b). At 2.5 mgFe/kg, there was a consistent linear decrease in blood pool SIR. However, at 5 mgFe/kg, the SIR initially remained constant before decreasing after 1.5 h. The highest tested concentration, 10 mgFe/kg, started with the lowest initial SIR, which then increased after 1.5 h.

In this study, a biphasic decrease over time in the blood pool concentration of ESIONs was noted, as measured by radioactivity. Time-dependent changes in MRI signal intensity showed varying patterns at different ESIONs concentrations. Specifically, a linear decrease in blood pool signal was noted at a low concentration (2.5 mgFe/kg) of ESIONs. In contrast, at medium (5 mgFe/kg) and high (10 mgFe/kg) concentrations, there was an initial signal plateau followed by a decrease, and a reduced signal, respectively. The alterations in signal intensity in T1-weighted MRI are attributed to a balance between the contrast-enhancing effect of T1 and the contrast-reducing effect of T2 [37]. Typically, signal quenching, due to the dominant T2 effect, is noted at higher concentrations [37, 51, 52], though this is infrequent in standard gadolinium-based T1-weighted MRI scenarios except in areas of high gadolinium concentration, like the bladder [53]. Iron oxide nanoparticle (IONP)-based contrast agents, exhibiting stronger magnetic properties than gadolinium, lead to a pronounced T2 shortening effect, and potentially causing significant signal reduction at clinical dosages. A phantom study with ultrasmall superparamagnetic iron oxide (USPIO) indicated a signal reduction at concentrations exceeding 0.2 mM in 3D T1-weighted GRE sequences [54]. Consequently, the optimal ESIONs dosage may vary based on the specific objectives of the study and the timing of image acquisition. Low concentrations are ideal for assessing concentration changes within the blood pool, while a medium concentration is optimal for sustained enhancement of the blood pool signal, facilitating accurate diagnostic imaging and characterization of vascular abnormalities.

### Toxicity of micelle encapsulated ESIONs

The results of the Trypan Blue exclusion assay indicate a dose-dependent decrease in HepG2 cell viability with increasing concentrations of ESIONs (Supplementary Fig. 5). Notably, significant cytotoxicity was observed above 960 ug/ml, indicating that ESIONs can induce hepatotoxic effects at elevated doses.

Preclinical toxicological studies in rats demonstrated that a single intravenous injection of up to 25 mg/kg was well tolerated and no deaths were observed. Results from the preclinical toxicity study is summarized in the Supplementary Table 2. There were no observed effects attributable to the micelle-encapsulated ESIONs on body weight, food intake, ophthalmic health, or urine output. Initial findings highlighted a temporary increase in neutrophils and decrease in lymphocytes at the highest dosage (25 mg/kg), suggesting a possible acute immune response, which was not evident in longer-term observations. Hematological and clinical biochemistry parameters remained unchanged in the primary test groups compared to controls.

Autopsy findings revealed no significant variations in organ weights across different groups. Although early histological evaluations noted Kupffer cell hypertrophy and hyperplasia at doses of 5 and 25 mg/kg, these were not deemed toxicologically significant in the comprehensive tests, with no abnormalities detected at injection sites. In our assessment of the toxicity profile for micelle-encapsulated ESIONs, we identified two critical observations requiring nuanced interpretation. Initially, there were noticeable yet temporary shifts in hematological parameters — specifically, an increase in neutrophils and a decrease in lymphocytes at a dosage of 25 mg/kg, noticeable only shortly after administration (2 days). These changes suggest a possible acute immune reaction to the introduced ESIONs. Additionally, early histological evaluations indicated Kupffer cell enlargement and proliferation within the liver at dosages of 5 and 25 mg/kg. Given that Kupffer cells are integral to the liver’s immune response, their brief activation could reflect a short-lived hepatic response to the micelle-encapsulated ESIONs. Notably, these alterations did not persist, with no significant changes in either hematological markers or liver conditions in the main test group after two weeks.

Iron oxide nanoparticles (IONPs), known for their excellent biocompatibility, are generally recognized as safe and minimally toxic. Historical research, tracing back to the earliest clinical applications, has aimed to delineate IONPs’ side effects in humans [55–57]. These studies generally report adverse events in only 5–24% of cases, with the majority being mild to moderate and severe adverse events being exceedingly rare. In a recent large-scale study involving 8666 patients treated with Ferumoxytol for iron deficiency, the incidence of side effects was recorded at 1.25%, with severe reactions noted in only 0.21% of cases [58]. In this context, the short-lived changes in hematological metrics and Kupffer cell activation observed in our study hint at a minimal long-term toxicity risk associated with ESIONs, aligning with the established safety profile of IONPs. Comprehensively, the findings from this study support the conclusion that micelle-encapsulated ESIONs exhibit a favorable safety profile for clinical application.

## Conclusions

In this study, we established an optimal protocol for scale-up process of micelle-encapsulated ESIONs by leveraging Tween 60 at a 10% v/v concentration, enabling straightforward surface modification. The high-capacity hydrophilic conversion using micelle-encapsulation in the g-scale conditions demonstrated enhanced stability against agglomeration in blood-like conditions. The promising r1 relaxivity and low r2/r1 ratio of the micelle-encapsulated ESIONs underscore their potential as effective T1 contrast agents. Furthermore, the biodistribution studies revealed a beneficial pharmacokinetic profile characterized by prolonged blood circulation and swift hepatobiliary clearance, where nearly 40% is excreted within 24 h. The safety profile is further validated by preclinical toxicity studies in rats, confirming their tolerability even at high doses. Collectively, these attributes position micelle-encapsulated ESIONs as a promising T1 contrast imaging agent with versatile clinical applications.

### Electronic supplementary material

Below is the link to the electronic supplementary material.

Supplementary Material 1

## Data Availability

The data supporting this study will be available upon request.
