# Supplementary material for: Optimization of micelle-encapsulated extremely small sized iron oxide nanoparticles as a T1 contrast imaging agent: biodistribution and safety profile
Source: J Nanobiotechnology. 2024 Jul 16;22:419. doi: 10.1186/s12951-024-02699-8 (PMC11253436; doi:10.1186/s12951-024-02699-8)
Supplement: Supplementary file 1 — Supplementary Material 1 [file 12951_2024_2699_MOESM1_ESM.docx]

**Supplementary Figure 1.**

**a.** Separation of nanoparticles in a density gradient using Iodixanol (Opti-prep) **b.** Schematic flow of hydrophilization method using NOTA-C18 for ESIONs.


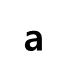

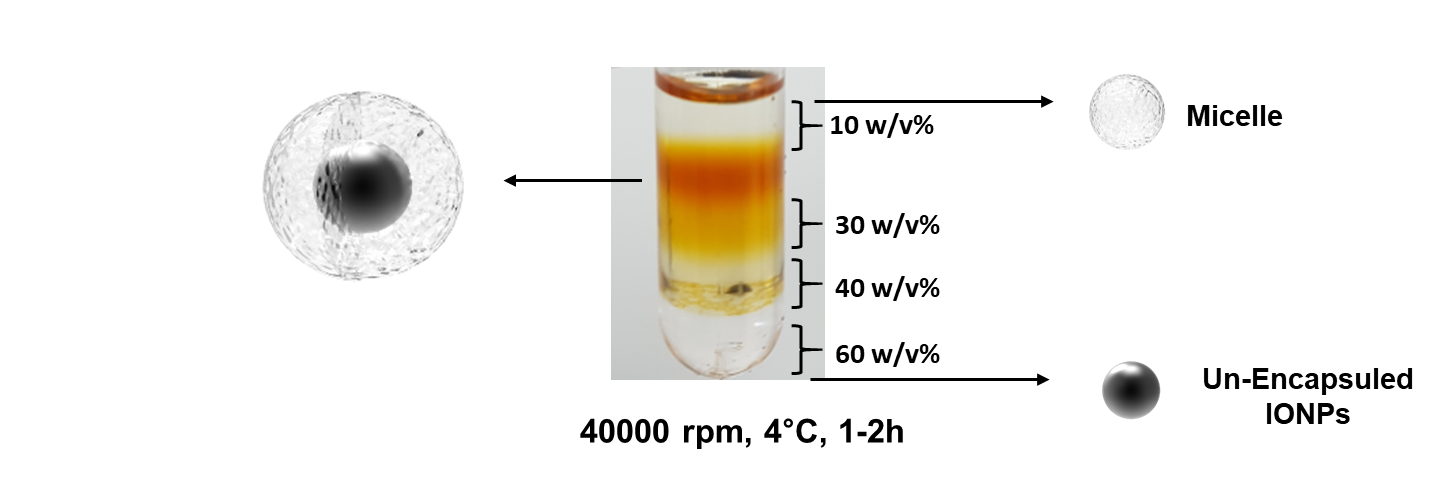


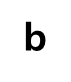


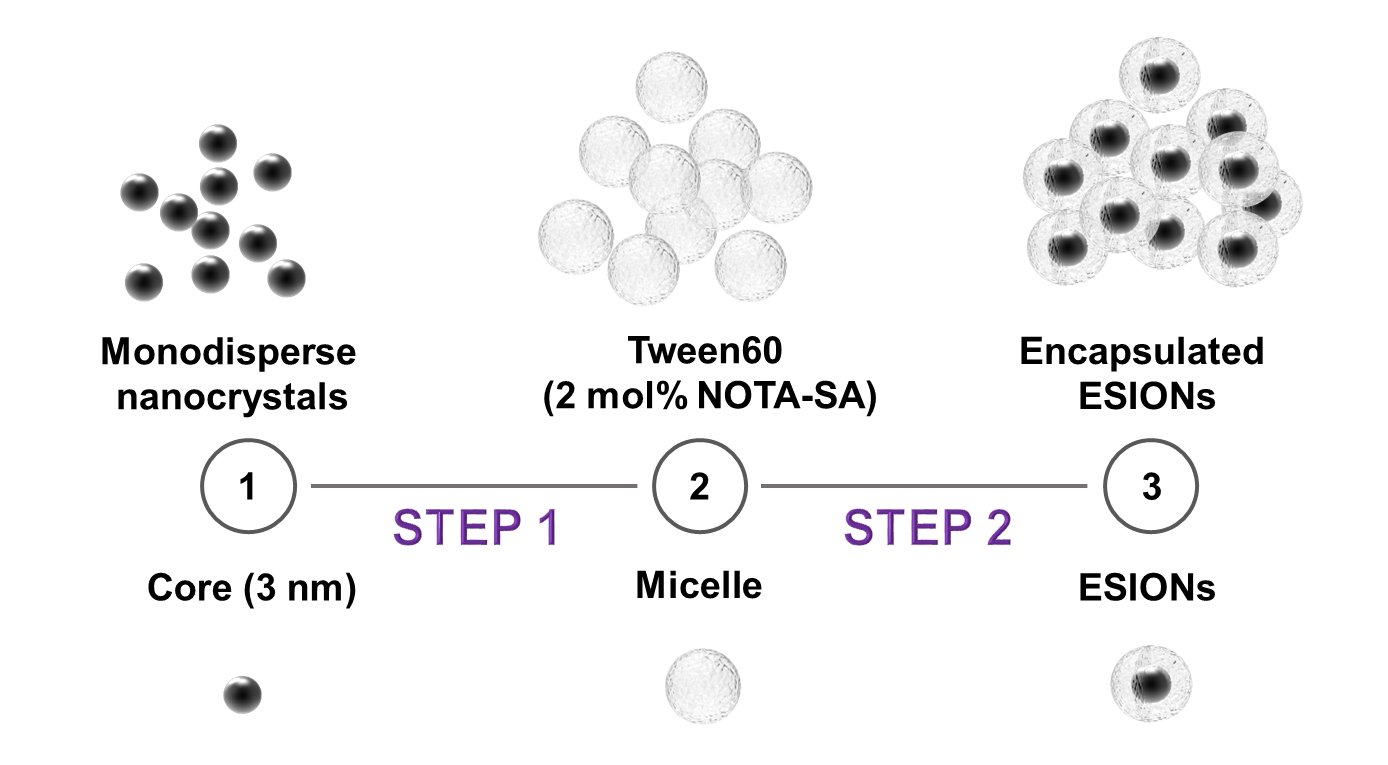


**Supplementary Figure 2.**

**a**, The shape and visual size of the ESIONs core were not changed on TEM image after micelle encapsulation. **b**, The hydrodynamic diameters of the micelle, micelle encapsulated ESIONs (baseline) and 64Cu-ESIONs (post-label) were 7.48 ± 1.51 nm, 9.35 ± 1.28 nm, and 9.47 ± 2.20 nm, respectively. **c**, After radiolabeling, r1 value (2.83 s-1mM-1) was estimated to be low compared to that of baseline (3.43 s-1mM-1). However, no significant change in the r2/r1 ratio was observed after radiolabeling (baseline vs post-label, 5.76 vs 5.36).

**
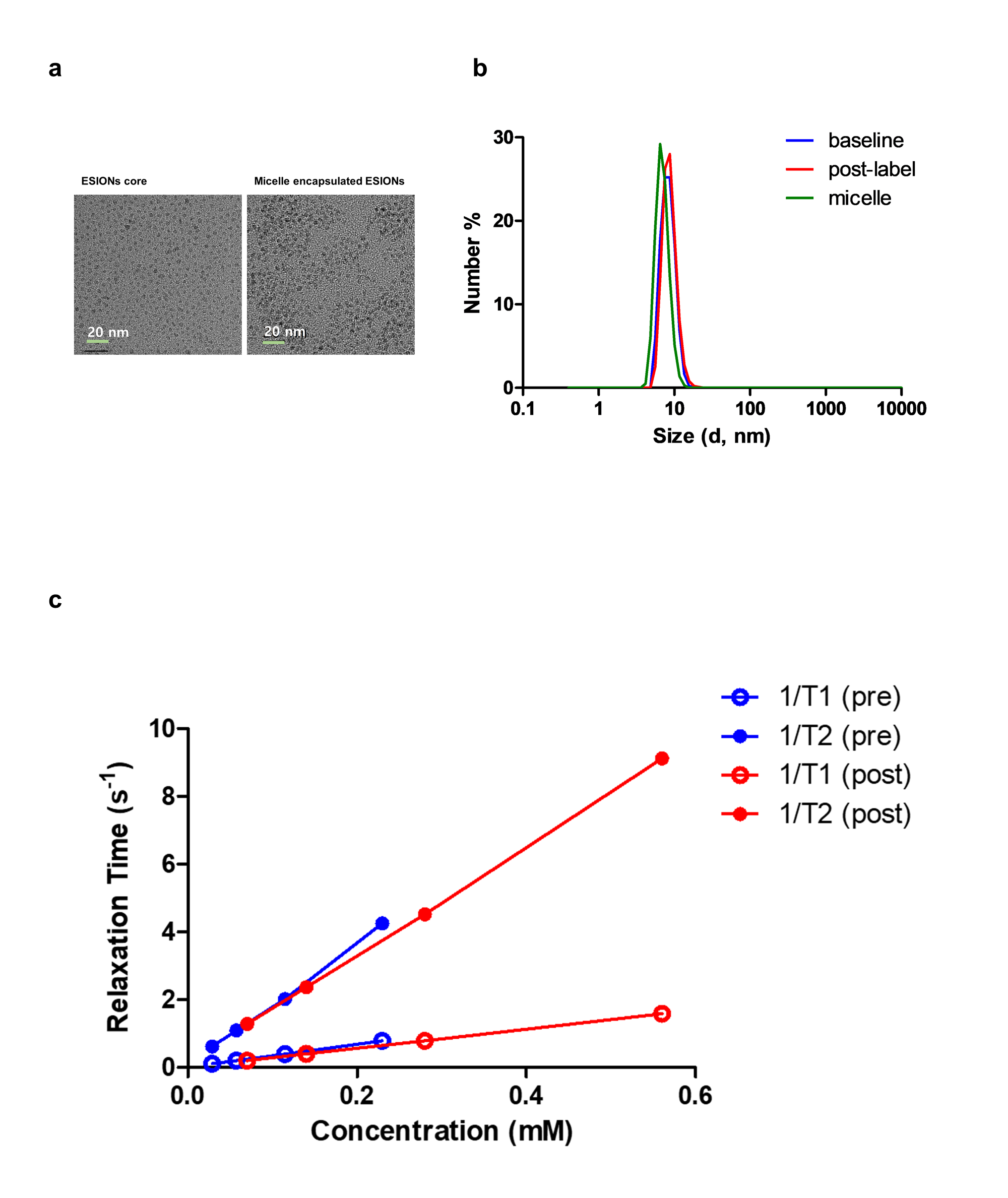
**

**Supplementary Figure 3.**

Labeling efficiency of ^64^Cu-ESIONs was over 95% and remained stable until 24 hours in PBS solution at room temperature (92.4%) and in human serum at 37 °C (77.1%).


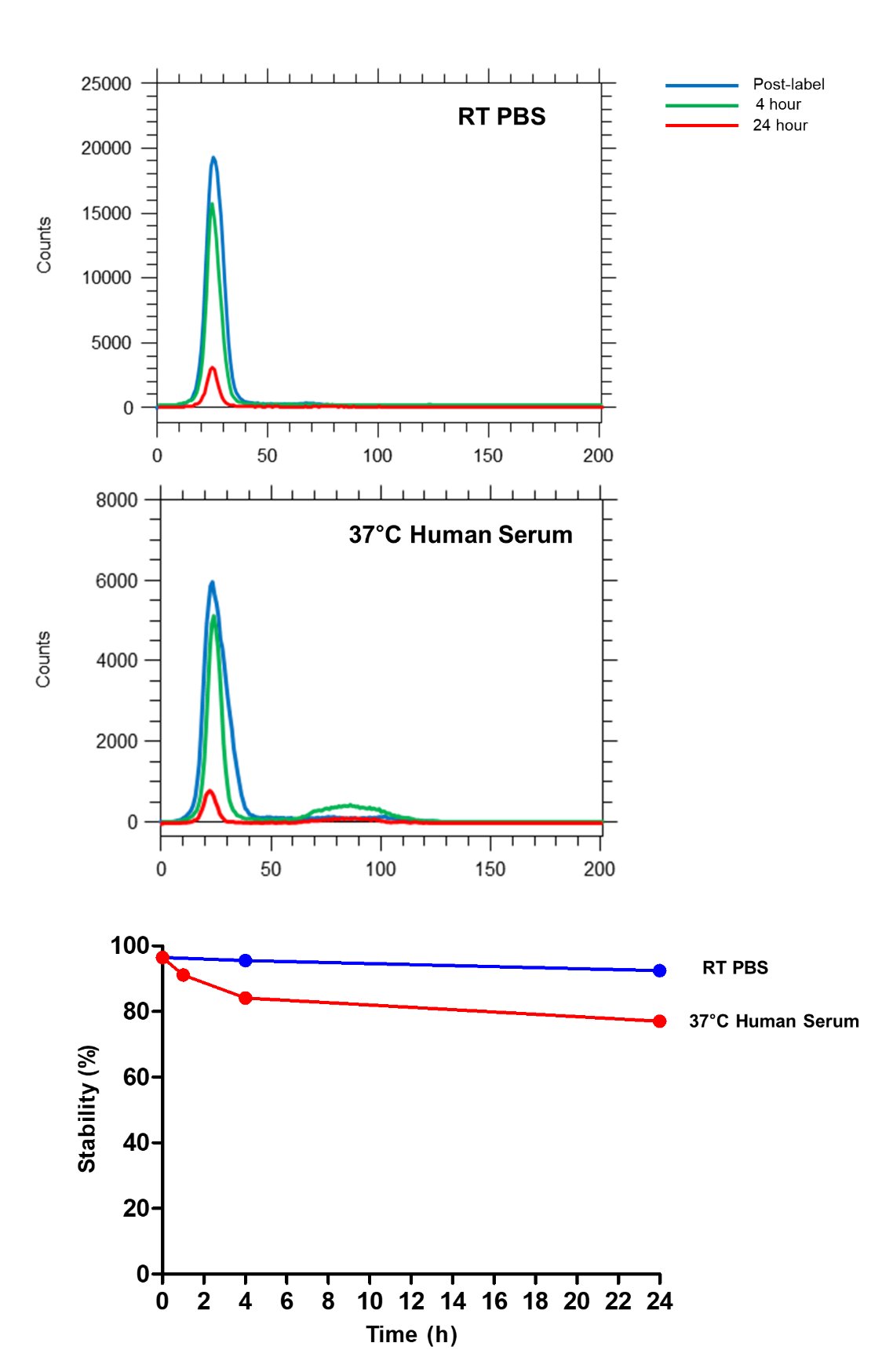


**Supplementary Figure 4.**

**a**, A blood sample was collected at different time points (5 min, 1 hour, and 4 hours) after administration of ^64^Cu-ESIONs. Radioactivity of blood was mostly detected as an intact form. **b**, A stool sample was collected, 4 hours after administration of ^64^Cu-ESIONs. The mixture of stool samples showed contrast enhancement in T1-weighted MRI with corresponding radioactivity. Radioactivity of blood was mostly detected as an intact form with radiochemical purity of 81%. **c**, A urine sample was collected, 1 hour after administration of ^64^Cu-ESIONs. Radio-thin-layer chromatography revealed than radioactivity of urine was mostly detected as a disintegrated form.

**
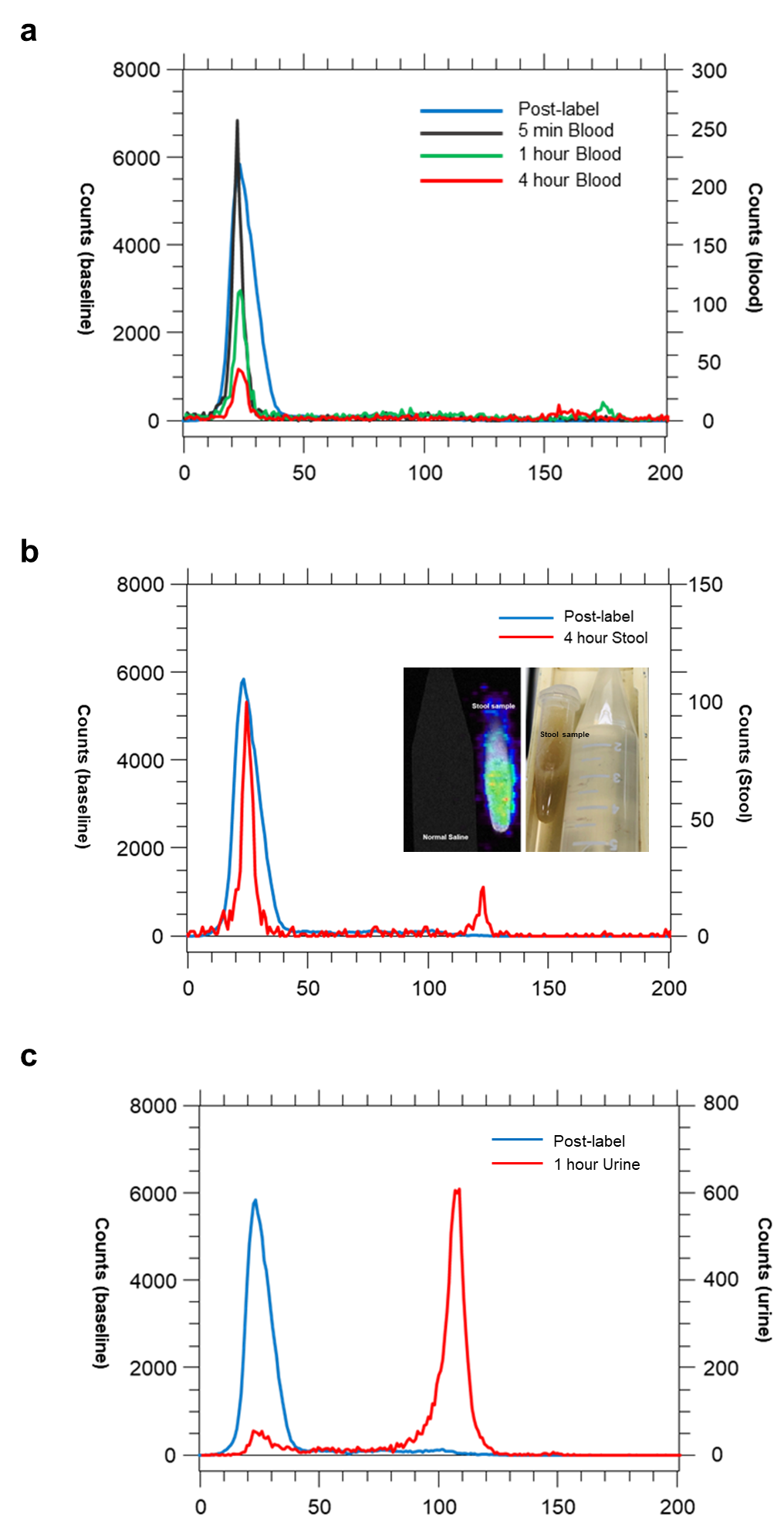
Supplementary Figure 5**

Viabilities of HepG2 cell following 24 hours exposure to ESIONs.

**
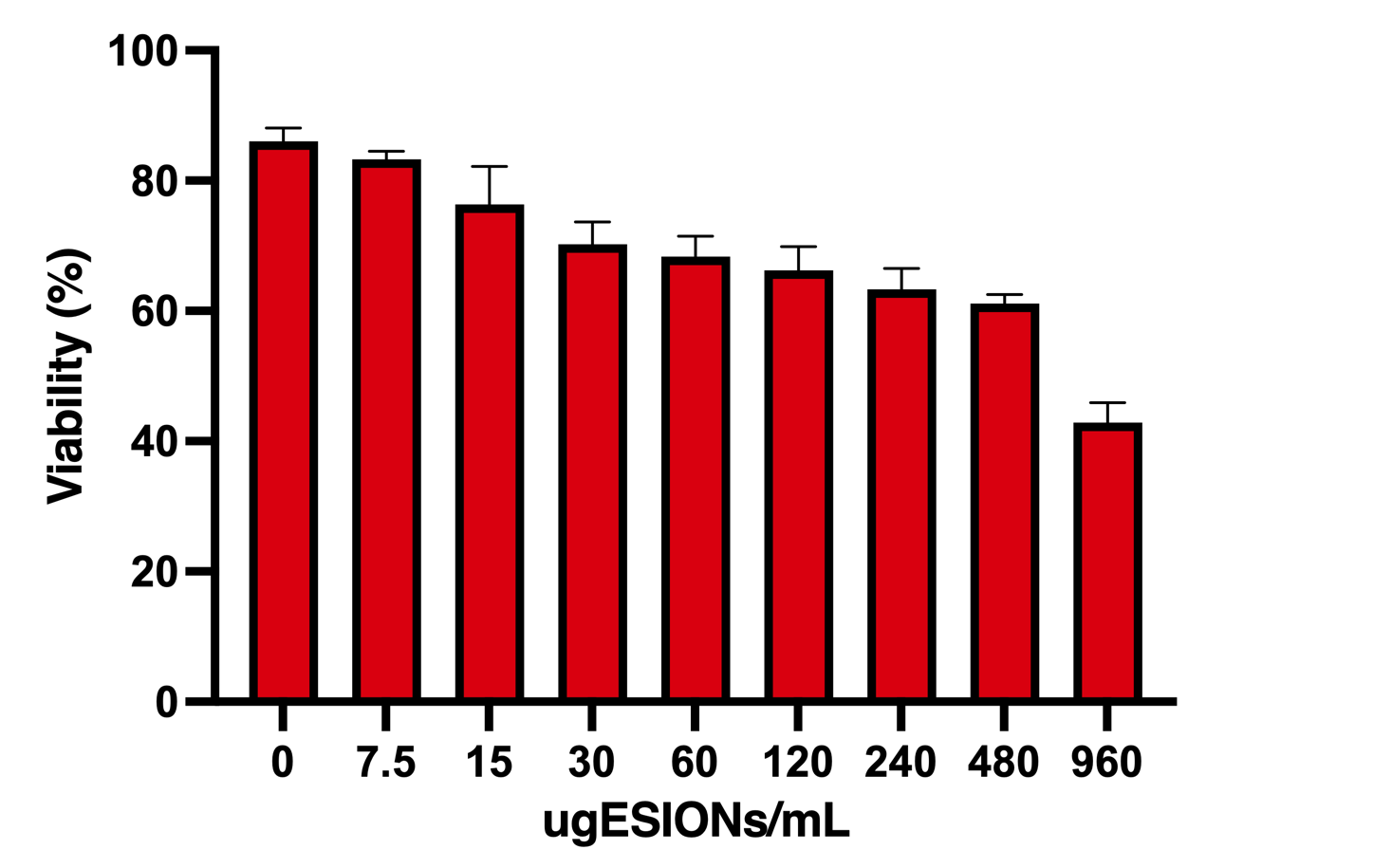
**

**Supplementary Table 1.**

| **Time** | | **5 min** | **1 hour** | **4 hours** | **24 hours** |
| --- | --- | --- | --- | --- | --- |
| **Organs** | Blood | 40.55 ± 1.98 | 15.98 ± 2.28 | 7.19 ± 1.03 | 0.74 ± 0.33 |
|  | Heart | 0.75 ± 0.08 | 0.34 ± 0.03 | 0.21 ± 0.02 | 0.09 ± 0.06 |
|  | Lung | 5.35 ± 1.01 | 2.79 ± 1.37 | 0.90 ± 0.58 | 0.47 ± 0.10 |
|  | Liver | 23.06 ± 2.96 | 36.63 ± 8.22 | 27.68 ± 1.99 | 24.18 ± 1.71 |
|  | Spleen | 1.06 ± 0.22 | 0.44 ± 0.17 | 0.27 ± 0.06 | 0.50 ± 0.16 |
|  | Stomach | 0.44 ± 0.14 | 1.21 ± 0.33 | 0.44 ± 0.23 | 0.36 ± 0.11 |
|  | Intestine | 3.21 ± 0.33 | 8.36 ± 1.49 | 13.81 ± 5.14 | 13.73 ± 1.92 |
|  | Kidney | 3.18 ± 0.38 | 2.22 ± 0.26 | 1.16 ± 0.17 | 1.28 ± 0.25 |
|  | **Total** | 77.61 ± 4.58 | 67.97 ± 13.12 | 51.66 ± 4.10 | 41.37 ± 2.93 |
| **Excreta** | Feces |  |  | 1.43 ± 1.18 | 37.34 ± 3.11 |
|  | Urine |  |  | 0.07 ± 0.02 | 4.38 ± 2.33 |

**Supplementary Table 2**

**Hematological parameters**

Interim sacrifice group (group 1, 0 mg/kg; group 2, 2.5 mg/kg; group 3, 5 mg/kg; group 4, 25 mg/kg)

Main group (group 5, 0 mg/kg; group 6, 2.5 mg/kg; group 7, 5 mg/kg; group 8, 25 mg/kg)

**Clinical chemistry parameters**

Interim sacrifice group (group 1, 0 mg/kg; group 2, 2.5 mg/kg; group 3, 5 mg/kg; group 4, 25 mg/kg)

Main group (group 5, 0 mg/kg; group 6, 2.5 mg/kg; group 7, 5 mg/kg; group 8, 25 mg/kg)

**Organ weights**

Interim sacrifice group (group 1, 0 mg/kg; group 2, 2.5 mg/kg; group 3, 5 mg/kg; group 4, 25 mg/kg)

Main group (group 5, 0 mg/kg; group 6, 2.5 mg/kg; group 7, 5 mg/kg; group 8, 25 mg/kg)
